# Conjunctival melanoma: comprehensive insights into clinical features, genetic alterations, and modern treatment approaches

**DOI:** 10.3389/pore.2025.1612085

**Published:** 2025-08-04

**Authors:** Snježana Kaštelan, Danijela Mrazovac Zimak, Luka Ivić, Antonela Gverović Antunica, Tamara Nikuševa-Martić

**Affiliations:** ^1^ Department of Ophthalmology, School of Medicine University of Zagreb, University Hospital Dubrava, Zagreb, Croatia; ^2^ Department of Ophthalmology, University Hospital Centre Zagreb, Zagreb, Croatia; ^3^ Department of Ophthalmology, General Hospital Dubrovnik, Dubrovnik, Croatia; ^4^ Department of Biology and Genetics, School of Medicine, University of Zagreb, Zagreb, Croatia

**Keywords:** conjunctival melanoma, clinical features, genetic alterations, metastasis, immunotherapy

## Abstract

Conjunctival melanoma (CoM) is a rare and aggressive ocular surface malignancy, characterised by increasing incidence, clinical complexity, and substantial challenges in diagnosis and treatment. This review consolidates current knowledge on epidemiology, clinical presentation, genetic and epigenetic foundations, molecular mechanisms, emerging therapeutic strategies, and prognostic factors for localised and metastatic CoM. CoM exhibits distinct biological behaviours, sharing molecular traits with cutaneous and mucosal melanomas, while significantly diverging from uveal melanoma. Key genetic alterations include mutations in BRAF, NF1, and PTEN, elevated mTOR expression, and specific miRNA profiles, which influence tumour progression and response to therapy. Recent advances in treatment, especially immune checkpoint inhibitors such as CTLA-4 and PD-1 receptor inhibitors, along with targeted therapies like BRAF and MEK inhibitors, have led to marked improvements in outcomes for advanced cases. Emerging strategies, including dendritic cell vaccines and epigenetic therapies, hold considerable promise in addressing ongoing clinical challenges. This review integrates case studies and clinical research to demonstrate the practical application of these therapies, highlighting their efficacy and limitations. Combining clinical expertise, genetic insights, and the latest therapeutic developments, offers a comprehensive overview of CoM, underscoring the critical role of a multidisciplinary approach in optimising diagnosis, management, and prognosis to improve patient outcomes.

## Introduction

Advances in oncology have improved our molecular and cellular understanding of cancer, leading to improved diagnosis, treatment, and the introduction of new therapies [1–3]. In parallel, considerable advancements in the treatment of melanoma have also been recorded in recent years [4].

Conjunctival melanoma (CoM) is a rare yet aggressive primary malignancy affecting the ocular surface [1, 3, 5]. It represents 5% of ocular melanomas and about 0.25% of all melanoma cases. The condition is most prevalent among individuals of European descent and has increased in incidence in recent decades [6–22]. It originates from malignantly transformed melanocytes in the conjunctival epithelium [7, 23]. Histopathologically, molecularly, genetically, and in terms of biological behaviour and management, CoM exhibits greater similarities to other mucosal as well as cutaneous melanomas (CM) than to uveal melanoma (UM) (Table 1) [1–9, 19, 21–44, 46, 47]. Furthermore, studies indicate that the incidence of CoM also varies geographically and is likely influenced by genetic and environmental factors [1, 3, 10, 19].

**TABLE 1:** Clinical and biological characteristics of melanoma types.

	Conjunctival melanoma	Cutaneous melanoma	Uveal melanoma	Other mucosal melanoma	References
Origin	Melanocytes in the basal conjunctival epithelium	Melanocytes in the epidermal basal layer	Melanocytes in the uveal stroma	Mucosal melanocytes (e.g., sinonasal, anorectal, vulvovaginal)	[1–5]
Incidence	0.3–0.8/100.000	19.7/100.000	2–6 per 1.000.000 (Europe); lower in Asia	1.5–2.8/1,000,000	[6–9, 19, 23–25]
UV Light as a Risk Factor	Probable	Well-established	Unclear	Not significant	[7, 21, 26–34]
Metastatic Pattern	Lymphatic and hematogenous spread (e.g., lymph nodes, liver)	Lymphatic and hematogenous spread (e.g., skin, lung, liver, brain)	Primarily hematogenous (liver, lung, bone)	Lymphatic and hematogenous spread	[7, 35–37]
Standard Treatment	Surgical excision ± adjuvant therapy (topical chemotherapy, cryotherapy, brachytherapy)	Surgery ± immunotherapy ± radiotherapy	Radiotherapy (brachytherapy) or enucleation; systemic therapy limited	Surgery ± immunotherapy ± radiotherapy	[1, 3, 22, 38, 39]
Immunotherapy Response	Under investigation; limited data	Responsive (anti-CTLA-4, anti-PD-1)	Limited efficacy	Variable, often less responsive	[40–44]
Genetic Alterations	BRAF V600E mutations (∼30%), NRAS mutations (∼20%), KIT mutations (exons 11, 13)	BRAF V600E mutations (∼50%), NRAS (∼20%), NF1 loss (∼15%)	GNAQ (∼50%) and GNA11 (∼40%) mutations; BAP1 mutations associated with metastasis	KIT mutations (∼25–40%), NRAS mutations (∼15–20%), occasional BRAF mutations (∼5–10%)	[4, 6, 37, 45, 46]
Chromosomal Alterations	Gains in 6p, 8q; losses in 6q	Gains in 1q, 6p, 7, 8q; losses in 9p21 (CDKN2A locus), 10q	Monosomy 3; gains in 8q; losses in 1p, 6q	Complex karyotypes; frequent losses in 3p, 6q, 10q; gains in 8q	[4, 6, 37, 45, 46]
Epigenetic Alterations	Promoter hypermethylation of RASSF1A, MGMT, p16 (CDKN2A)	Global DNA hypomethylation; promoter hypermethylation of CDKN2A, PTEN, RASSF1A	BAP1-associated chromatin remodeling defects; hypermethylation of RASSF1A, p16 (CDKN2A)	Aberrant DNA methylation of tumor suppressors (e.g., CDKN2A, RASSF1A); altered histone acetylation (decreased H3K27ac)	[4, 6, 37, 45, 46]

UV: ultraviolet; CNS: central nervous system; CTLA-4: cytotoxic T-lymphocyte-associated antigen 4; PD-1, programmed cell death-1.

Melanomas generally demonstrate varied behaviours, genetic characteristics, and responses to treatment. Significant therapeutic strides have been made in managing CM, particularly with targeted therapies and immune checkpoint inhibitors (ICIs). In contrast, progress in treating CoM has been limited by the lack of established treatment protocols, a shortage of clinical trials, and a limited understanding of the immunology of ocular tumours and their microenvironment [37].

The primary treatment for localised CoM typically involves operative removal combined with additional treatment, including cryotherapy, brachytherapy, chemotherapy, or immunotherapy [2, 37, 45]. Despite these approaches, the high recurrence rate of up to 66% following surgical excision with adjuvant therapy highlights the need for more effective treatment options. There is currently no universally accepted standard therapy for metastatic CoM, thus, treatment is often adapted from protocols used for CM [21, 38]. The introduction of molecular inhibitors and immunomodulatory therapies has improved the treatment of metastatic CoM [37, 45]. Additionally, depending on the location, some isolated metastases can be treated with surgical resection or radiation therapy, which have also demonstrated some success in treating metastases in UM patients. While evidence regarding targeted therapies and immunotherapy for CoM is still limited, existing case reports and series suggest these approaches may be effective for managing recurrent, locally advanced, and metastatic CoMs [1–3, 9, 26, 43–46, 48–55]. Several molecular studies have uncovered genetic and epigenetic alterations linked to CoM that may help elucidate its metastatic potential [56]. As with any cancer, deepening the knowledge regarding the molecular and genetic processes driving CoM development, progression, and metastasis may help to identify novel predictive biomarkers and treatment targets, potentially improving treatment results for these patients [9].

This paper aims to provide a comprehensive overview of recent advancements in the genetic, biological, immunological, and clinical aspects of CoM and to evaluate their implications for prognosis and treatment strategies.

## Clinical and biological characteristics of conjunctival melanoma

### Epidemiology

CoM is a rare ocular malignancy, representing 2%–5% of all ocular tumours and 5%–7% of all ocular melanomas. Its incidence rate in the individuals of European descent adult population is 0.3–0.8 per million [5, 10, 12]. Compared to individuals of European descent, black people and Asians have a significantly lower incidence of conjunctival melanoma (CoM) [18]. Several studies have shown that the CoM incidence rate is rising [5, 18]. The incidence of the condition increases with age, with the average age at clinical presentation ranging from 55 to 65 years, and a mean age of 57.4 years at histopathological diagnosis [21]. It is exceedingly rare in the population under the age of 20 [5, 21, 34, 47]. While no definitive gender predilection has been identified, some studies have shown that males tend to be younger than females when diagnosing primary tumours [5, 21, 47].

### Aetiology

CoM can arise *de novo* or from pre-existing melanocytic lesions, most commonly conjunctival melanocytic intraepithelial lesions (C-MIL), which account for approximately 70% of cases [2, 57, 58]. Previously termed conjunctival melanocytic intraepithelial neoplasia (C-MIN) or primary acquired melanosis (PAM) with atypia, C-MIL represents a preinvasive spectrum ranging from melanocytic hyperplasia to melanoma *in situ* [59]. In 2018, the fourth edition of the *WHO Classification of Ocular Tumours* introduced a simplified grading system that categorized C-MIL as low-grade (corresponding PAM with mild or no atypia or C-MIN grades 1–2), high-grade (corresponding PAM with moderate to severe atypia or C-MIN grades 3–5), and melanoma *in situ* (PAM with severe atypia involving >75% of the epithelial thickness or C-MIN >5). This system was validated in 2021, demonstrating comparable predictive accuracy across the C-MIL, C-MIN, and PAM classifications for recurrence risk [57].

In 2022, the fifth edition of the classification refined this scheme, acknowledging that the previous low-grade C-MIL category encompassed both neoplastic and non-neoplastic melanocytic proliferations. The current system stratifies C-MIL into low- and high-grade categories based on histopathologic features. Low-grade C-MIL is characterized by predominantly basal melanocytic proliferation with mild cytologic atypia and carries a relatively low risk of progression to invasive melanoma. In contrast, high-grade C-MIL exhibits basal and prominent suprabasal proliferation of atypical melanocytes, marked cytologic atypia and a significantly higher risk of invasive transformation. Notably, melanoma *in situ* is now included within the high-grade C-MIL category, referring to lesions with near full-thickness epithelial involvement or those that histologically resemble melanoma without evidence of subepithelial invasion [59]. The revised classification, validated in a large international study, showed strong interobserver agreement, high reproducibility, and prognostic value, supporting its use in guiding therapy [58, 59].

Conjunctival melanocytic nevi are common benign proliferations of melanocytes, typically forming in the first decade of life. Histopathologically, the three most common types are junctional nevi, compound nevi, and subepithelial nevi, which may represent different stages of melanocyte maturation and proliferation [60]. Although conjunctival nevi rarely undergo malignant transformation, approximately 2% of cases can develop into melanoma [61]. Nonetheless, about 7% of all CoMs are believed to originate from pre-existing conjunctival nevi [47].

Deep penetrating nevi (DPN), also known as melanocytomas, account for 9.4% of all excised conjunctival nevi. Defined by their distinctive morphology, DPNs exhibit a nested or plexiform growth pattern of primarily epithelioid melanocytes with vesicular nuclei and finely pigmented cytoplasm, often accompanied by melanophages. Immunohistochemical analysis typically shows positivity for the BRAFV600E mutation, with activation of the beta-catenin pathway frequently observed. Clinically, conjunctival DPNs appear as dark brown pigmented lesions with uniform or irregular pigmentation, most commonly found on the bulbar conjunctiva (44%), caruncle (21%), and semilunar fold (21%). Due to their atypical clinical features and growth potential, these lesions are often excised. Accurate recognition of DPN of the conjunctiva is essential to prevent its misdiagnosis as melanoma, given that DPN is a benign lesion [62–65]. Additionally, in 11%–26% of cases, CoMs develop “*de novo*,” with no precursor lesions being identified [20, 21, 47, 66].

### Clinical presentation

CoMs typically present as asymptomatic raised pigmented plaques, tumours, or macules on the bulbar or tarsal conjunctiva [6]. The most commonly affected sites are the bulbar conjunctiva (56%–79% of cases), the conjunctiva of the fornices and palpebrae (9%–29% of cases), and the caruncle (1%–7% of cases) [66–68]. While these tumours are often pigmented, they can also be non-pigmented or show mixed appearance [47, 67, 68]. Although multiple lesions are uncommon, they have been reported more frequently in cases associated with PAM [47].

### Histopathology

Histologically, CoM comprises various cell types, including nevoid, epithelioid and spindle cells. Nevoid cells resemble benign nevi. Epitheloid cells are large with abundant cytoplasm and prominent nucleoli, showing significant pleomorphism and mitotic activity, while spindle cells are elongated with less cytoplasm. Tumor architecture varies, presenting as flat, nodular, or diffuse growths, sometimes with intraepithelial spread. Deeper tissue invasion, such as into the sclera or orbit, indicates advanced disease and a worse prognosis. Although variable, melanin pigmentation is a notable feature, and thus heavily pigmented melanomas are easier to diagnose, while amelanotic melanomas require immunohistochemical (IHC) staining for identification. IHC markers, including S-100 protein, HMB-45, Melan-A, and SOX10, confirm the melanocytic origin of the tumour and distinguish it from other pigmented lesions [20, 26, 32].

### Risk factors

Exposure to ultraviolet (UV) radiation is a well-established risk factor for CM. However, its role in the development of CoM remains a topic of debate [6]. Epidemiological studies have suggested a correlation between the increasing incidence of CoM and decreasing latitude, indicating that sun exposure may play a role in its development [25, 26]. Despite these findings, the exact impact of UV radiation on CoM is not yet fully understood.

Several studies have documented the presence of a UV signature in DNA damage from CoM samples [26, 29, 30]. A recent study revealed that 86% of bulbar CoMs exposed to sunlight exhibited a high (>70%) mutational load of C > T changes, indicative of UV-induced DNA damage. CoMs in sun-exposed bulbar areas more frequently harbour BRAF mutations than those from non-exposed sites [20]. BRAF mutations are found in about one-third of CoMs, with the V600E mutation being the most prevalent, present in approximately 80% of cases [20, 26, 32]. These mutations are associated with intermittent sun exposure, suggesting a potential link between UV exposure and CoM [33]. However, other studies have found no significant difference in the expression of oncology-related genes between melanomas from sun-exposed and non-exposed areas [69].

Several conditions are associated with an increased risk of CM, including familial atypical multiple mole melanoma (FAMMM) syndromes and BAP-1 tumour predisposition syndrome [70]. However, up to the present time, no similar conditions have been identified as risk factors for CoM.

## Genetic alterations in conjunctival melanoma

### Overview of genetic studies

Most genetic studies on CoM primarily analyse somatic mutations and structural variations in primary tumour samples. This focus is due to the sporadic nature of CoMs, employing targeted or comprehensive methods. CoM exhibits a unique genetic profile that overlaps significantly with mucosal and cutaneous melanomas, but less with UM. Key mutations in the CoM landscape include alterations in genes such as BRAF, NRAS, KIT, NF1, and ATRX, which often coexist with UM-associated mutations like BAP1, SF3B1, and GNAQ/11. These genetic alterations are correlated with advanced disease, an increased risk of metastasis, and poorer prognosis, indicating a need for proactive treatment approaches and rigorous monitoring for affected patients [71].

### Key signaling pathways

Two highly complex and interconnected biological pathways commonly deregulated in CoMs are:

1. Mitogen-Activated Protein Kinase (MAPK) Pathway: Also known as the RAS/RAF/MEK/ERK pathway, it regulates gene expression by converting numerous genes into RNA, sending growth signals to the nucleus, and controlling multiple cellular activities such as differentiation, proliferation, and survival [45].
2. Phosphatidylinositol 3-Kinase (PI3K)/AKT/mTOR Pathway: This pathway is also intricately linked to tumour formation through the overactivation of proto-oncogenes and the inactivation of tumour suppressor genes [45].

The presence of a “UV mutational signature” characterised by CC > TT substitutions and a predominance of C > T substitutions at dipyrimidine sites indicates DNA damage from UV light. This signature often corresponds with a higher tumour mutational burden (TMB), reflecting differences between epithelium-associated melanomas (such as cutaneous and mucosal melanomas) and non-epithelium-associated melanomas (like uveal and leptomeningeal melanomas) [72]. Mucosal melanomas, including CoM, typically show a lower TMB and fewer UV signals, despite being more common in sun-protected areas. Ocular melanomas that arise in varying sunlight exposure conditions demonstrate similar UV signature presence and TMB levels, with CoMs often having higher TMB levels linked to UV exposure [73].

### Key mutations in conjunctival melanoma

The gene BRAF, which encodes a serine/threonine kinase that activates the MAPK pathway by triggering MEK, is situated on chromosome 7 at the q34 region. Certain oncogenic mutations in BRAF cause the BRAF proteins to become activated on their own, permanently activating MEK1/2 and ERK1/2 via the MAPK pathway and promoting the formation of tumours [45]. Roughly one-third of CoMs have been reported to contain BRAF mutations [32]. While mutations can arise at other codons of the BRAF gene, the majority of documented mutations have occurred at codon 600, where valine is replaced by glutamic acid (p.V600E; 80%–90%), lysine (p.V600K; 9%–20%), or infrequently by another amino acid. These features resemble CMs, although posterior UMs typically do not have BRAF mutations [74, 75]. BRAF-mutated CoMs occur more frequently on sun-exposed/bulbar conjunctiva, suggesting UV exposure as a potential risk factor [20, 76].

Situated on chromosome 1p13, NRAS belongs to the same family as other RAS genes. It codes for a GTPase incorporated into the MAPK cascade and upstream of BRAF. It may also be the first step in the PI3K/AKT/mTOR pathway [56]. While NRAS mutations are uncommon in posterior UMs, they were found in 20% of the CoMs, similar to CM [74]. Point mutations in the NRAS gene that affect codons 61 (Q61R and Q61K are the most common) or codons 12 or 13 (G12/13) lead to uncontrolled cell division [56]. Conjunctival nevi also show NRAS mutations [77]. A link between NRAS mutations and more aggressive tumour features, including a higher chance of metastasis and death, has been suggested [71, 76]. MEK inhibitors have been studied as single medicines or in conjunction with PI3K/mTOR inhibitors for tumours with NRAS mutations, although data regarding their application in advanced CoM with NRAS mutation is lacking [78].

Chromosome 17q11 contains the NF1 gene, which produces a tumour suppressor protein that prevents RAS and acts as an inhibitory regulator of the PI3K/AKT/mTOR and MAPK pathways. Higher RAS activity is linked to loss-of-function or inactivating NF1 mutations, which lead to excessive signaling. NF1 mutations have been detected in about one-third of CMs, mostly nonsense or frameshift mutations. Although rare, F1 mutations can coexist with NRAS or BRAF mutations in CoMs [71, 76]. There is no known correlation between NF1 mutations and clinicopathological characteristics or prognosis [71, 76]. Like CMs, NF1 mutations seem more common in CoMs linked to a UV signature, indicating potential benefits from immunotherapy for patients with NF1 mutations [9, 31, 48].

Chromosome 4q12 contains the KIT gene, which encodes a receptor tyrosine kinase [RTK] that activates several downstream pathways, including the PI3K/AKT/mTOR and MAPK pathways [56]. BRAF and NRAS mutations are typically absent from CoMs with activating mutations and/or gains in the KIT gene/locus, indicating mutual exclusivity [76]. KIT mutations can coexist with NF1 mutations in CoMs, similar to the way BRAF and NRAS mutations can. KIT mutations are frequently found in non-sun-exposed CMs and sun-protected mucosal melanomas. Although no correlation has been observed between CoM survival and KIT status, c-KIT inhibitors are appropriate targets for KIT-mutated malignancies, although their effectiveness in CoM patients remains unclear [78].

The PTEN gene, located on chromosome 10q23, encodes a tumour suppressor protein that inhibits the AKT/mTOR pathway by negatively regulating PI3K. Loss of PTEN activity, due to mutations, deletions, or decreased expression, leads to increased PI3K activity and excessive PI3K/AKT/mTOR signaling. Like skin melanomas, CoMs may exhibit elevated mTOR pathway activity and decreased or absent PTEN expression. Notably, PTEN expression is generally higher in UMs than in CoMs [9, 79].

The cellular location of PTEN (nuclear versus cytoplasmic) influences its activity, with the nuclear fraction primarily responsible for tumour suppression. CoMs show more prominent nuclear PTEN loss than conjunctival nevi, suggesting a significant role in oncogenesis and malignant transformation. Recent studies have reported inactivating PTEN mutations alongside copy number changes that induce PTEN loss in CoMs. Although PTEN and NRAS mutations typically do not co-occur, they frequently appear with BRAF or KIT mutations [78].

Interestingly, a study linked PTEN loss to CoM pigmentation, indicating that amelanotic tumours exhibited greater nuclear PTEN expression than pigmented tumours. Despite the lack of correlation with other CoM-related characteristics or prognosis/survival thus far, CoMs with PTEN loss may be candidates for targeted treatments using mTOR inhibitors [71].

### Telomere maintenance

The TERT gene, located on chromosome 5p15, encodes the catalytic protein subunit of telomerase, a ribonucleoprotein polymerase that maintains telomere length. In normal somatic cells, telomerase expression is suppressed, leading to telomere shortening and eventual cell senescence. However, abnormal telomerase activity can allow cells to become “immortal.”

Like skin melanomas, CoMs typically contain 35%–40% TERT promoter (TERTp) mutations at similar sites [78]. These mutations often exhibit a characteristic UV signature and can co-occur with BRAF or NRAS mutations. TERTp mutations can enhance TERT expression, allowing neoplastic cells to survive indefinitely, although the exact causes of elevated TERT expression in CoMs remain unclear. While conjunctival nevi do not have TERTp mutations, lesions with atypia do, suggesting a link to malignant transformation. Recent findings indicate that TERTp mutations are also present in non-PAM-derived CoMs, warranting further investigation. Unlike CoMs, TERTp mutations are uncommon in posterior UMs, but they have been associated with metastatic development in CoMs, highlighting their prognostic significance [74]. Furthermore, TERTp-mutated cancers may eventually be treated with telomerase and reverse transcriptase inhibitors [9, 80].

### Chromatin remodeling

The ATRX gene, located on chromosome Xq21, encodes a chromatin remodelling protein essential for homologous recombination and DNA methylation-mediated epigenetic regulation of alternative telomere lengthening (ALT). Inactivating mutations and loss of ATRX protein expression are frequently observed in malignancies utilising the ALT pathway for telomere maintenance, such as mucosal melanomas [81].

ATRX mutations have been identified in approximately 20%–60% of CoM patients, with subsequent validation confirming these mutations in 25% of cases. Functional studies revealed that ATRX-mutated tumours exhibit ALT positivity and loss of ATRX protein expression [71]. ATRX mutations co-occur more frequently with NF1 mutations than NRAS or BRAF mutations. Additionally, ATRX-mutated CoMs often harbour mutations in genes associated with histone modification and epigenetic regulation, such as HDAC, SETD genes, CREBBP, or MLLT6 [9, 48].

ATRX mutations also frequently co-occur with TP53 alterations in CoMs and other mucosal melanomas. While ATRX loss and TERT activation typically demonstrate mutual exclusivity in various cancers, further research is needed to explore their combined genetic changes in CoMs. The early detection of ATRX loss and ALT positivity in both the intraepithelial and invasive components of CoMs suggests their involvement in tumorigenesis. The prognostic relevance of ATRX-mutated CoMs is reflected in their tendency to develop in non-sun-exposed areas and their association with less aggressive behaviour. CoMs with ATRX mutations may also resist anti-telomerase therapy while being vulnerable to PARP inhibitors, indicating potential therapeutic implications [71]. In their study, van Ipenburg et al. report a correlation between TERT promoter mutations and decreased metastasis-free survival in conjunctival melanoma (CoM). The findings indicate that CM with ATRX loss also tends toward poorer outcomes, highlighting that both TERT promoter mutations and ATRX loss are associated with adverse clinical behaviour. The presence of TERT promoter mutations was strongly linked to shorter metastasis-free survival, suggesting a similar risk profile for CM cases exhibiting ATRX loss [82]. Additional genes found in CoM are presented in Table 2.

**TABLE 2:** Recent studies utilising targeted next-generation sequencing or unbiased whole genome/exome sequencing have identified various mutations in CoMs.

Gene	Chromosomal location	Function	References
ATM	11q22	Cell cycle checkpoint kinase regulating multiple proteins	[76]
TP53	17p13	Tumor suppressors involved in various cellular processes	[31]
CDKN2A	9p21	Tumor suppressor proteins that control the cell cycle	[69]
FBXW7	4q31	Involved in the degradation of oncoproteins	[48]
TET2	4q24	Methylcytosine dioxygenase important for epigenetic control	[83]
SETD2	3p21	Histone methyltransferase involved in epigenetic regulation	[48]
IDH1	2q34	Important in metabolism	[31]
CBL	11q23	E3 ubiquitin ligase interacting with signaling proteins	[83]
ALK, MET	2p23 (ALK), 7q31 (MET)	Tyrosine kinase receptors	[76]

Furthermore, other mutated genes relevant to CoM pathophysiology have been identified, including CTNNB1, ACSS3, PREX2, APOB, RYR1/2, SYK, NOTCH3, CHEK2, KMT2A/C, ARID2, FAT4, RB1, APC, and members of the MAPK/MAP2K/MAP3K signaling cascades. Their precise roles remain to be clarified and merit further investigation [84].

### Chromosomal aberrations

CoMs also display various chromosomal abnormalities, including.

• Numerical chromosomal abnormalities: Polyploidy or aneuploidy.
• Gains: Notable regions include 1p, 3p, 6p, 7p/q, 8p/q, 11p/q, 12p, 13q, 14p, 17q, and 22q.
• Losses: Include regions such as 1p, 3q, 4q, 6q, 8p, 9p/q, 10p/q, 11q, 12q, 15p, 16p/q, 17p, 19p/q, and 21p [25, 27–29, 35, 70, 85].

Amplifications in regions like 6p21–25, particularly at 6p22’s histone cluster 1 area, suggest the presence of important oncogenic drivers (e.g., BRAF, NRAS, and TERT) while deletions affecting NF1, TP53, and others indicate a complex genetic landscape [25, 31, 70].

Despite the unclear processes underlying recurrent chromosomal aberrations in CoMs, integrative analyses could provide insights. Patterns of CNAs vary with genetic backgrounds, with BRAF/NRAS wild-type tumours showing notable increases [86].

### Epigenetic hallmarks

MicroRNAs (miRNAs) play a significant role in CoM pathophysiology by facilitating post-transcriptional gene silencing. Many miRNAs, such as miR-30d, miR-506, miR-509, miR-146, and miR-20b, are elevated in CoM and may serve as therapeutic targets or prognostic indicators. For instance, upregulation of miR-20b is associated with PTEN suppression, and inhibiting miR-506 and miR-509 reduces cell proliferation and invasiveness in CoM [24, 87].

Understanding the interactions of miRNAs, such as miR-146a with NOTCH proteins, emphasises their role in early cancer formation in CM and highlights potential avenues for targeted therapies in CoM management [88, 89].

Key findings from the study by Larsen et al. (2016) identified specific miRNAs distinctly expressed in conjunctival melanoma compared to healthy conjunctival tissue. These miRNAs may help differentiate malignant tissue from normal conjunctiva, aiding in diagnosis. Several miRNAs, such as miR-204 and miR-211, were found to be significantly downregulated in conjunctival melanoma. This downregulation was associated with more aggressive tumour characteristics, suggesting these miRNAs could serve as prognostic biomarkers for assessing the risk of tumour progression. The dysregulated miRNAs are involved in pathways critical for cancer development, including cell proliferation, apoptosis, and immune response modulation. These pathways are essential in understanding the mechanisms behind conjunctival melanoma’s aggressive behaviour [89].

Mikkelsen et al. (2019) identified unique miRNA expression patterns in metastatic conjunctival melanoma, with certain miRNAs overexpressed in metastatic cases compared to non-metastatic samples. Specific miRNAs, such as miR-21 and miR-146b, were notably associated with metastatic behaviour in conjunctival melanoma. These miRNAs may have potential as prognostic biomarkers, helping to identify patients with a higher risk of metastasis. Understanding miRNA involvement in metastasis offers potential therapeutic targets, as manipulating miRNA levels could provide a new approach to slow disease progression and improve patient outcomes in metastatic conjunctival melanoma [13]. Also study by van Ipenburg et al. (2020) identified five miRNAs that were upregulated in conjunctival melanoma compared to nevi, with higher levels of miR-9-5p, miR-196b-5p, and miR-615-3p strongly associated with malignancy. The shared pathway involving these miRNAs, possibly linked to homeobox gene clusters, suggests a role in conjunctival melanoma pathogenesis. Additionally, this miRNA combination may help distinguish benign from malignant lesions, especially when tissue samples or diagnostic methods are limited. However, no miRNAs were identified to predict metastatic potential, underscoring the need for further research in this area [90].

With advancements in RNA sequencing and bioinformatics, circular RNAs (circRNAs), a type of circular non-coding RNA, have emerged as a focal point in cancer research [91]. Numerous circRNAs linked to cancer have been identified by various research teams, highlighting their potential roles in tumour development and progression. In the study of Shang et al. (2019), the authors identified over 9,300 circRNA candidates in conjunctival melanoma tissue compared to adjacent normal tissue. Among these, circMTUS1 was confirmed as a circular RNA upregulated in melanoma tissues and cell lines. Functional assays demonstrated that circMTUS1 supports tumorigenesis both *in vitro* and *in vivo*, likely by sequestering hsa-miR-622 and hsa-miR-1208 and influencing pathways associated with cancer. This suggests that circMTUS1 may serve as a novel biomarker for conjunctival melanoma, providing potential diagnostic and therapeutic targets in this field [92].

## Prognostic insights

CoM is a highly aggressive cancer with a strong tendency for both local recurrence and metastatic spread [21, 93–95]. This dual threat not only endangers vision but also poses a significant risk to life, highlighting the necessity for thorough insight into its pathogenesis for improving clinical management and treatment outcomes.

CoMs possess a local and systemic metastatic potential with an overall mortality rate of approximately 30%. The metastatic disease occurs in 20%–30% of cases, with the tumour cell spreading through the lymphatic system and hematogenous [6, 37, 96]. In 45%–60% of cases, metastases are initially found in the regional lymph nodes, including ipsilateral preauricular, submandibular, parotid, and cervical lymph nodes [47, 97]. Systemic spread most commonly occurs in the brain, lungs, liver, skin, bones, and gastrointestinal tract [21, 37, 67, 68, 98, 99]. The local recurrence rate is notably high, ranging from 30% to 62%, and is associated with a worse prognosis [6, 21, 37, 47, 68]. Factors that increase the risk of local recurrence include tumours located in non-epibulbar sites (such as the palpebral conjunctiva, fornices, and eyelid margins), surgical excision performed alone without adjuvant therapy, and tumour excisions with histopathologically unclear margins [6, 21, 47, 66, 68, 96, 99].

The 5-year survival rate for CoM is approximately 86.5%, while the 10-year survival rate, depending on various factors, ranges from 41% to 78% [21]. Poor prognostic indicators for CoM include patient age under 55 years, melanomas extending beyond one quadrant with a diameter greater than 10 mm, tumour thickness exceeding 2 mm, multifocal tumour presentation, nodular tumour appearance, histopathological findings of atypical or mixed cell melanocytes with a lack of inflammatory response, and local tumour recurrence [21, 47, 66–68, 99].

Although the prognosis may improve with new targeted therapy and ICIs, current prognostic data for larger patient groups remain limited, with most evidence coming from case reports [37].

The tumour’s BRAF status does not correlate with prognosis, whereas mutations in the TERT promoter gene have prognostic implications [96]. While BRAF mutations may not currently influence prognosis, they could become significant as BRAF/MEK inhibitors may be used to treat metastatic disease, similar to their application in CM [100]. TERT promoter mutations, associated with prognosis, could also shape future therapeutic strategies [80]. Although the incidence of CoM in children and adolescents is low and the literature on these cases is limited [96]. The available data suggests that the survival rate for children is generally more favourable than that of adults [34].

The latest 8th edition of the American Joint Committee on Cancer (AJCC) TNM classification system offers a comprehensive classification for CoM, detailing tumour (T), node (N), and metastasis (M) stages [101, 102]. In the previous 7th edition, higher T grades (T2, T3, T4) were associated with a significantly increased local recurrence rate, regional lymph node metastasis, distant metastasis, and mortality [103]. The 8th edition was validated through a large multicenter international study involving 288 eyes from 288 patients with CoM. This study confirmed that higher clinical tumour categories (cT2 and cT3 vs. cT1) and pathological tumour categories (pT2 and pT3 vs. pT1) correlated with elevated mortality rates. Additionally, tumour thickness, ulceration, and invasion were identified as independent prognostic factors for increased mortality risk, while the involvement of the caruncle or plica did not show a significant association [23, 102, 104].

The TNM classification provides an accurate tool for disease staging. Higher T categories, lymph node involvement, and distant metastases are strongly linked to poorer prognoses, highlighting their important role in risk stratification. This stratification enables personalised treatment planning by guiding decisions on surgical interventions, adjuvant therapies, and surveillance strategies. Precise staging of the disease allows clinicians to identify patients who may benefit from aggressive interventions such as SLNB, systemic or immune therapies, or enrollment in clinical trials. Additionally, the TNM classification ensures appropriate treatment intensity, avoiding overtreatment in early-stage cases while identifying high-risk patients requiring more aggressive management. The TNM system also provides a standardised framework for reporting and comparing clinical outcomes across studies and institutions. This consistency facilitates collaborative research and advances evidence-based practices in the management of CoM [94, 102].

Additional histopathological features correlated with worse disease prognosis include survival tumour thickness, surgical margin involvement, predominantly epithelioid cell type, ulceration, lymphovascular invasion, necrosis, high mitotic rate, and microsatellite lesions [6, 35, 105].

## Treatment strategies for conjunctival melanoma

Treatment modalities for CoM are primarily determined by the tumour’s location and extent of spread. Localised disease is treated by surgical excision with adjuvant therapy, including cryotherapy using a “double freeze-thaw” technique, topical chemotherapy (mitomycin-c drop or interferon-alpha), and radiotherapy [45, 47, 106]. On the other hand, the treatment of metastatic disease poses a significant clinical challenge, as there is currently no standardised therapeutic protocol for the treatment of metastatic disease in patients with CoM [39].

### Localised disease treatment

The preferred treatment for localised CoM involves a comprehensive approach that includes total surgical excision using a “no-touch” technique. This method employs new, clean instruments at every stage of the procedure, reduces the possibility of tumor seeding, and requires excision-wide tumor-free conjunctival margins of 2–4 mm. Supplemental cryotherapy using a “double freeze-thaw” technique is applied to the conjunctival margins, and alcohol corneal epithelialectomy is performed if the tumour extends to the corneal limbus. It is important to preserve the Bowman layer, as it serves as a natural barrier against tumour invasion [6]. Supplemental treatments aim to eliminate any clinically undetectable tumour cells that may remain along the resection margins, thus preventing the spread of viable tumour cells [68]. Surgical excision alone, without adjuvant therapies such as plaque brachytherapy, topical chemotherapy (*e.g*., mitomycin C), or interferon alpha-2b, is generally discouraged due to the higher risk of local recurrence and increased mortality [21, 45, 68, 99].

Several prospective and retrospective series have confirmed that combining wide local excision and cryotherapy with adjuvant topical chemotherapy or plaque brachytherapy significantly improves outcomes in patients with localized CoM. In a long-term study involving 85 patients, Werschnik and Lommatzsch reported a 10-year tumor-related survival rate of 77.7% and an overall survival rate of 62.5%. Notably, they observed significantly fewer recurrences in patients who received adjunctive treatment, such as irradiation, cryotherapy, or local chemotherapy with mitomycin C (MMC), in addition to surgical excision, compared to excision alone [99]. Similarly, a large nationwide cohort study conducted in the Netherlands, encompassing 194 patients treated between 1950 and 2002, found a local recurrence rate of 58% (median follow-up of 6.8 years) and a regional lymph node metastasis rate of 21%. Outcomes were significantly improved in patients treated with adjuvant brachytherapy compared to those who underwent excision alone or excision with cryotherapy [21]. In a cohort of 150 patients, Shields et al. demonstrated that the absence of adequate adjuvant therapy was associated with a 26% metastasis rate at 10 years and a tumor-related mortality rate of 13% by 8 years [68].

Cryotherapy applied to the surgical margins following excision plays a crucial role in eliminating residual tumor cells, with its mechanism of action involving both direct cytotoxic effects, such as disruption of cellular integrity through intracellular content efflux, and ischemic injury resulting from damage to the local microvasculature [107]. The adjunctive use of cryotherapy has been shown to significantly reduce the risk of tumor recurrence compared to excision alone. Specifically, recurrence rates have been reported at 18% with adjuvant cryotherapy versus 52% with excision alone [108, 109]. These findings underscore the importance of incorporating cryotherapy into the standard surgical management of CoM to improve local disease control and reduce recurrence rates in CoM.

Topical chemotherapeutic agents used as adjuvant therapy for CoM include mitomycin C (MMC) and interferon alpha-2b. MMC, an alkylating agent, is the most commonly used agent and is considered the standard adjuvant therapy in many centers. To reduce the risk of scleral thinning or melting, initiation of therapy is typically delayed for several weeks following surgical excision, allowing for sufficient wound healing. MMC is usually administered at a concentration of 0.04%, four times daily, in treatment cycles lasting one to 3 weeks, separated by 1-week drug-free intervals. Although its efficacy as a primary treatment is limited due to poor penetration through the basement membrane and reaching deeper tissues, MMC effectively eliminate residual superficial tumor cells. Topical application is frequently associated with transient but often severe keratoconjunctivitis, which is self-limiting and occurs in nearly all patients [110–112]. In a phase I trial by Finger et al., adjuvant MMC (0.04% QID for 7 days following excision) resulted in no tumor recurrence over a mean follow-up period of 29 months [106].

Interferon alpha-2b, a naturally occurring cytokine with antiproliferative, immunomodulatory, and pro-apoptotic effects, represents an alternative adjuvant approach. It exerts its antitumor activity by prolonging the cell cycle, enhancing the expression of tumor suppressor genes, and downregulating oncogene expression [113]. Administered topically at a concentration of 1,000,000 IU/mL, four to five times daily for six to 12 weeks, interferon alpha-2b is generally well tolerated and may be particularly beneficial for patients who are intolerant to MMC. However, its role in the treatment of CoM remains fully elucidated, and further prospective studies are needed to establish its efficacy [114, 115].

Radioactive plaque brachytherapy represents a well-tolerated and effective adjuvant modality in the multidisciplinary management of CoM. While CoM exhibits relative radioresistance and plaque brachytherapy is not typically employed as a primary treatment, its adjuvant use offers a distinct advantage by delivering localized radiation to deeper stromal tissues, beyond the reach of topical chemotherapeutic agents. Ruthenium-106 plaques are most frequently utilized, delivering a prescribed dose of 100 Gray to a standardized depth of 2 mm. This targeted approach has demonstrated favorable local control rates, with reported recurrence rates of 19% at 3 years and 21% at 5 years, while preserving visual function and minimizing ocular morbidity [116, 117]. These outcomes support the integration of plaque brachytherapy into the treatment algorithm for select CoM patients, particularly those with high-risk histopathological features or residual deep scleral invasion following surgical excision.

Incisional biopsies should generally be avoided due to the risk of tumour spread and local recurrence [68, 118]. However, they may be considered in cases where total surgical removal of the tumour is not feasible [6]. Orbital exenteration is reserved for patients with extensive CoM involving orbital or intraocular invasion [66]. Sentinel lymph node biopsy (SLNB) is recommended for melanomas larger than 10 mm in diameter, and 2 mm in thickness, with histological ulceration, scleral invasion, or tumors found in areas other than the bulbar conjunctiva [6, 119–123]. It offers an early opportunity for intervention before systemic metastasis occurs and can detect subclinical nodal metastases missed by clinical or ultrasound examination [119, 124]. Typically performed after excision of the primary tumour, SLNB can be important for accurate staging and guiding treatment decisions. A positive SLNB is associated with poorer metastasis-free and disease-specific survival, underscoring its importance for prognosis and identifying high-risk patients for adjuvant therapy [104, 125]. While SLNB offers valuable prognostic information in selected patients with CoM, certain contraindications and technical challenges may limit its broader application. Prior surgeries or radiation in the head and neck may alter lymphatic drainage, impairing SLN localization. Hypersensitivity to radiotracers or dyes, significant comorbidities, and minimal metastatic risk, such as *in situ* or thin (<1 mm) tumors, further restrict its indication. The periocular region presents unique challenges, including the need for precise tracer injection near critical structures and the risk of technetium leakage, which can be reduced by immediate ocular coverage and contralateral head positioning. Ophthalmic administration and preoperative lymphoscintigraphy improve accuracy while maintaining low radiation exposure. Facial nerve injury during parotid dissection and transient blue staining of ocular tissues highlights the need for specialized surgical expertise. Despite these considerations, SLNB remains a safe and informative procedure when applied within established protocols [5, 98, 104, 121, 126].

### Metastatic disease treatment

#### Targeted molecular inhibitors

Targeted therapy selectively disrupts oncogenic pathways by influencing specific genetic mutations in malignant cells, sparing healthy tissues. In contrast to conventional chemotherapy, it reduces systemic toxicity by focusing on cancer-specific molecular mechanisms [45, 50, 56, 127–130]. Most CoMs harbour mutations within the MAPK pathway, involving genes such as *BRAF*, *RAS*, *c-KIT*, and *NF1.* [56] Inhibitors targeting BRAF (vemurafenib, dabrafenib) and MEK (trametinib, cobimetinib) have shown efficacy in MAPK-driven melanomas and are used in both cutaneous and conjunctival subtypes [1, 4, 39, 45, 56].

Combined BRAF/MEK inhibition improves treatment efficacy and delays resistance more effectively than monotherapy [50, 113]. However, responses in CoM may be less effective than in cutaneous melanoma due to resistance mechanisms, including *PTEN* loss and MAPK pathway reactivation [127, 128, 131, 132].

A major challenge with BRAF inhibitor monotherapy is the development of resistance, which often occurs within a year of initiating treatment. Resistance mechanisms include the upregulation of NRAS, NF1, or ERK, and the downregulation of PTEN [127, 128, 131, 132]. Combining BRAF and MEK inhibitors has been more effective than BRAF inhibitor monotherapy alone [50, 129]. However, compared to their effectiveness in treating CM, BRAF inhibitors may be less effective for CoM due to frequent PTEN loss, which affects resistance.

Current insights into CoM treatment outcomes are based on small series and case reports (Table 3) [26, 43, 44, 48–55]. The main goal of systemic targeted therapy in CoM is to control extensive local disease that cannot be surgically excised or to serve as an alternative to orbital exenteration. These therapies are also designed to target regional and distant metastases, offering a more comprehensive approach to disease management [4, 133]. The dosing schedule of CoM therapy is equivalent to that of CM [134]. Additionally, there are cases where anti-PD-1 agents have been used in combination with targeted therapy, as documented by Dagi Glass (2017) and Kiyohara (2020) [43, 55].

**TABLE 3:** Reported cases of targeted molecular inhibitor therapy in locally advanced, recurrent or metastatic BRAF mutant conjunctival melanoma cases.

Author, year	Country	Type of study	Patient	Adjuvant treatment	Local treatment in the advanced stage	Systemic therapy in the advanced stage	Outcome (PFS/OS)
Primary conjunctival melanoma							
Pahlitzsch et al. (2014) [44]	Germany	Case report	Female 80y	Excision + brachytherapy (ruthenium)	Eyelid surgery after recurrence	vemurafenib	PR; stable for 3 years; OS not reported
Demirci et al. (2019) [48]	USA	Case series	Female 70y	None	Excision after systemic therapy	dabrafenib + trametinib	Regression after 3 months, local control; metastasis after 12 months
Kim et al. (2020) [49]	USA	Case report	Male 52y	None	Excision	dabrafenib + trametinib	CR at 10 months; metastasis-free at 15 months
Metastatic conjunctival melanoma							
Weber et al. (2013) [50]	USA	Case report	Male 45y	None	Resection	vemurafenib	PR at 1 month; PD at 2 months
Griewank et al. (2013) [26]	Germany	Case report	Male 43y	Resection + radiotherapy (ruthenium)	Proton therapy	dabrafenib	PR initially; PD at 6 months
Maleka et al. (2016) [51]	Sweden	Case report	Female 53y	Excision + cryotherapy + mitomycin C	Enucleation	vemurafenib	PR; PD after 4 months OS < 5 months
Pinto Torres et al. (2017) [52]	Portugal	Case series	Female 56y	Excision + electron beam radiotherapy	None	vemurafenib	CR at 1 month; OS ≥ 36 months
Demirci et al. (2019) [48]	USA	Case series	Female 70y	None	Excision after systemic therapy	dabrafenib + trametinib	Regression; no local recurrence; brain and lung metastases at 12 months
Rossi et al. (2019) [53]	Italy	Case report	Male 70y	Excisional biopsy	Parotidectomy + lymphadenectomy	dabrafenib + trametinib	PR; lymph node reduction
Kiyohara et al. (2020) [43]	Japan	Case series	Male 72y	Excision + cryotherapy + mitomycin C	None	dabrafenib + trametinib	CR; OS 6 months (alive and recurrence-free)
Miura et al. (2022) [54]	Japan	Case report	Female 89y	None	Resection	encorafenib + binimetinib	PR at 6 months; reduction of metastases
Combined therapy with immune checkpoint inhibitors and targeted molecular inhibitor therapy							
Dagi Glass et al. (2017) [55]	USA	Case report	Female 61y	Excision + cryotherapy	Parotidectomy and modified radical neck dissection	1: dabrafenib and trametinib 2: vemurafenib 3: pembrolizumab 4: vemurafenib 5: vemurafenib + cobimetinib	CR after 1 month OS ≥ 23 months
Kiyohara et al. (2020) [43]	Japan	Case series	Male 71y	Excision + Cryotherapy	Enucleation	1: vemurafenib 2: nivolumab 3: nivolumab + dabrafenib + trametinib	Died 24 months after combination therapy

PR: partial response; OS: overall survival; CR: complete response; PD: disease progression; PFS.

#### Immune checkpoint inhibitors

Immune checkpoint inhibitors (ICIs) enhance antitumor immunity by targeting regulatory pathways that tumors exploit to suppress immune responses [135]. These monoclonal antibodies block checkpoint proteins such as cytotoxic T-lymphocyte-associated antigen 4 (CTLA-4), programmed cell death-1 (PD-1), and programmed cell death ligand-1 (PD-L1), thereby restoring T-cell activation and promoting tumor cell elimination [3, 45, 85, 136–140]. ICIs have shown clinical efficacy across several melanoma subtypes, including CoM, with therapeutic responses influenced by factors such as TMB, a surrogate marker of immunogenicity [3]. In CoM, ICI regimens typically follow protocols established for cutaneous melanoma [137].

CTLA-4 functions as a negative regulator of T-cell responses. It inhibits T-cell activation by binding to CD80 and CD86 on antigen-presenting cells, thereby blocking the essential costimulatory signals. CTLA-4 inhibitors counteract immune suppression, such as ipilimumab (an IgG1 monoclonal antibody) and tremelimumab (an IgG2 monoclonal antibody) [137]. Targeting CTLA-4 has been shown to promote tumour rejection and enhance the development of immunologic memory. PD-1, a receptor expressed on T-cells, plays a role in downregulating the immune system and promoting self-tolerance. By binding to PD-L1 or PD-L2 on cancer cells, PD-1 inhibits T-cell activity. PD-1 inhibitors, such as nivolumab and pembrolizumab, have proven effective in treating metastatic CoM [138]. These inhibitors block PD-1, which enables T-cell activation and enhances the immune response against cancer cells [136]. Approximately 19% of CoMs express PD-L1, and this expression is linked to the presence of distant metastases and worse survival outcomes [85].

The molecular similarities between CM and CoM, and the expression of PD-1/PD-L1 in a subset of CoM, suggest that checkpoint inhibition could be a promising treatment option [6]. ICIs used in CoM treatment are ipilimumab, an anti-CTLA4 inhibitor, and nivolumab and pembrolizumab an anti-PD-1 inhibitor [1, 3, 4, 45, 56, 137, 139].

In CoM therapy, ICIs have shown more favourable outcomes than in UM, with responses ranging from partial response to complete regression. These inhibitors have proven effective in managing locally advanced and metastatic diseases [138, 141, 142]. Additionally, combined therapy with anti-CTLA-4 and anti-PD-1 agents produces a synergistic effect, enhancing outcomes in CoM treatment by downregulating multiple phases of T-cell activation [133]. However, data regarding this therapy is limited, with only a few case reports and case series exploring the use of ICIs for recurrent, locally advanced, and metastatic CoM (Table 4) [52, 138, 142–155].

**TABLE 4:** Reported cases of checkpoint inhibitor therapy in locally advanced, recurrent and metastatic conjunctival melanoma cases.

Author, year	Country	Type of study	Patient	Adjuvant treatment	Local treatment in the advanced stage	Systemic therapy in the advanced stage	Outcome
Primary conjunctival melanoma							
Kini et al. (2017) [143]	USA	Case report	Male 60y	Excision + cryotherapy	None	pembrolizumab	PFS 12 months; OS ≥ 12 months
Esmaeli et al. (2019) [144]	USA	Case report	Female 56y	None	None	nivolumab	PR; follow-up NR
Finger and Pavlick (2019) [142]	USA	Case series	Female 94y	None	None (Exenteration rejected)	1: pembrolizumab 2: pembrolizumab + ipilimumab	1) PD 2) PR; OS 5 months
			Male 76y	Multiple local treatments + topical IFN-α drops	None	1: ipilimumab 2: pembrolizumab 3: pembrolizumab + IFN- α	CR; PFS 36 months
			Female 84y	Excision + cryotherapy Mitomycin C Plaque brachytherapy	None	1: pembrolizumab + ipilimumab 2: pembrolizumab + ipilimumab + IFN- α	CR; PFS 36 months
Hong et al. (2021) [145]	USA	Case series	Female 53y	Mitomycin C 0.02%	None	1: pembrolizumab 2: pembrolizumab + mitomycin C	CR; PFS 12 months
Alhammad et al. (2022) [146]	Saudi Arabia	Case report	Female 32y	Excision + cryotherapy + mitomycin C	None	ipilimumab + nivolumab	CR; PFS 54 months
Attrash et al. (2024) [147]	Israel	Case report	Female 87y	None	None	nivolumab + relatlimab	None
Benchekroun Belabbes et al. (2025) [201]	USA	Case report	Male 55y	Excision + cryotherapy	Exenteration + lymphadenectomy	pembrolizumab + radiotherapy	PFS 12 months
Matsuo et al. 2022 [148]	Japan	Case report	Female 80 years	None	Proton beam therapy	pembrolizumab	Tumour regressed; died suddenly at 7 months
Weiss et al. 2025 [149]	USA	Case report	Male 59 years	None	None	ipilimumab + nivolumab	Local control at 7 months
Metastatic conjunctival melanoma							
Pinto Torres et al. (2017) [52]	Portugal	Case series	Male 51y	Multiple excisions	Lymphadenectomy	pembrolizumab	PFS 24 months
Sagiv et al. (2018) [138]	USA	Case series	Female 58y	Multiple resections + parotidectomy	Orbital exenteration	nivolumab	CR; follow-up 3 months
			Female 28y	Excision + cryotherapy + mitomycin C	None	nivolumab	PFS 36 months
			Female 47y	Excision + cryotherapy + radiotherapy + Parotidectomy + LND + IFN-α + Mitomycin C	Radiotherapy	nivolumab	CR; PFS 7 months
			Female 68y	Resection + Mitomycin C + Exenteration + SLNB + Parotidectomy + radiotherapy	Exenteration + Radiotherapy	1: pembrolizumab 2: ipilimumab + dacarbazine	1) PFS 6 months; then PD 2) PR ​
			Male 74y	Multiple excisions	None	nivolumab	PFS 1 month
Chaves et al. (2018) [150]	Brazil	Case report	Male 72y	Debulking + SLNB + I-125 brachytherapy + Neck dissection	I-125 brachytherapy	ipilimumab	CR; follow-up NR
Chang et al. (2019) [151]	USA	Case report	Female 60y	Excision + orbitotomy + cryotherapy + radiotherapy	Radiotherapy	1: ipilimumab + nivolumab 2: nivolumab 3: pembrolizumab	PR; PFS 24 months
Finger and Pavlick (2019) [142]	USA	Case series	Female 72y	Local excision + topical chemotherapy	None	ipilimumab + nivolumab	PR
			Female 76y	Excision + Cryotherapy + Topical mitomycin chemotherapy	Parotidectomy + surgery + radiotherapy	1: ipilimumab 2: ipilimumab 3: pembrolizumab	Ipilimumab-new skin metastases and lymph metastases. Pembrolizumab – OS 2y
Bay et al. (2020) [152]	Turkey	Case report	Male 13y	None	Palliative radiotherapy	1: temozolomide 2: ipilimumab	No response; OS 19 months
Poujade et al. (2020) [153]	France	Case report	Female 68y	Complete excision	None	pembrolizumab	CR; OS ≥ 24 months ​
Hong et al. (2021) [145]	USA	Case series	Male 66y	None	None	ipilimumab + nivolumab	CR at 9 months
Matsuo et al. (2022) [148]	Japan	Case report	Female 80y	None	Proton-beam therapy	pembrolizumab	CR at 7 months; died suddenly
Fan et al. (2023) [155]	USA	Case report	Female 60y	Excision + cryotherapy + radiotherapy	External beam radiotherapy	1: ipilimumab + nivolumab/4 cycles 2: nivolumumab	25% reduction; PFS >16 months; no recurrence at 1 year
Waninger et al. (2024) [154]	USA	Case series	Male 50y	Excision + cryotherapy	1) Parotidectomy + LND 2) Excision + cryotherapy + I-125 brachytherapy 3) Exenteration	1: ipilimumab 2: pembrolizumab 3: carboplatin + paclitaxel	Death at 6 years
Weiss et al. (2025) [149]	USA	Case report	Male 59y	None	None	ipilimumab + nivolumab	CR at 7 months

PFS: progression-free survival; PR: partial response; OS: overall survival; CR: complete response; PD: disease progression; LND: lymph node density; SLNB: sentinel lymph node biopsy.

Although ICIs can induce tumor regression, they may also trigger pseudoprogression, a transient increase in tumor size caused by immune cell infiltration rather than true disease progression [156]. This presents a clinical challenge in distinguishing between treatment response and actual progression [142, 143, 145, 150, 151]. Additionally, ICIs are associated with immune-related adverse events, as nonspecific T-cell activation can result in off-target inflammation and damage to healthy tissues [137, 138, 157–164].

#### Dendritic cell vaccination

DC vaccination is a personalized immunotherapy that harnesses autologous antigen-presenting cells to generate tumour-specific T-cell responses. Patient monocytes or haematopoietic progenitors are harvested by leukapheresis and differentiated *ex vivo* into immature DCs using GM-CSF and IL-4. These DCs are then loaded with tumour-associated peptides or cell lysates and reinfused. Following administration, DCs migrate to tumour-draining lymph nodes, mature, and cross-present antigen via MHC I/II to prime naïve CD8^+^ cytotoxic and CD4^+^ helper T cells. Clinical trials in metastatic CM report enhanced intratumoral CD8^+^ infiltration and significant prolongation of median overall survival. Although no human studies exist in CoM, a mouse model combining cDC2-subset vaccination with osteopontin blockade demonstrated marked anti-angiogenic activity and immune stimulation in early ocular melanoma. Such findings underscore the translational potential of DC vaccines across melanoma subtypes. Future investigations should optimize antigen selection, loading protocols, and adjuvant combinations to enhance vaccine efficacy [37, 41, 165–168].

#### Innovative immune-based approaches

Novel immune-based strategies for malignant melanoma focus on modulating the tumour microenvironment. One preclinical approach uses nanoparticles to co-deliver atovaquone and cabozantinib, aiming to reduce hypoxia and suppress immunosuppressive cells. This combination enhances anti-tumour immunity by improving T-cell activation in tumour-bearing mice [37, 56, 169]. While still experimental, such approaches represent a promising direction for future melanoma therapy development.

#### Epigenetic approaches

Epigenetic regulation, predominantly DNA methylation and histone acetylation/deacetylation, modulates gene expression without altering nucleotide sequences, thereby governing proliferation, drug sensitivity, and resistance. Aberrant methylation silences key tumour suppressors (RASSF1A, APAF1, CDKN2A, PTEN, TP53), while dysregulated histone modifications activate oncogenes (RAS, MDM2, MITF, ERK, c-JUN, BCL-2). Therapeutic agents include DNA methyltransferase inhibitors (decitabine) and histone deacetylase inhibitors (panobinostat). Decitabine induces DNA hypomethylation and re-expression of silenced genes; when combined with ipilimumab in inoperable melanoma, it upregulates HLA-I and expands intratumoral CD8^+^ PD-1 T cells and CD20^+^ B cells. In phase I trials of decitabine plus panobinostat and temozolomide, 75% of refractory metastatic melanoma patients achieved disease stabilization or complete response. Panobinostat also promotes chromatin relaxation, differentiation, and G1 arrest in UM models, reducing viable cell fractions. Emerging histone methyltransferase inhibitors and miRNA modulators further sensitize tumours to cytotoxic T and NK cells and enhance antigen presentation. To date, these epigenetic strategies remain untested in CoM [22, 130, 170–177].

#### Adoptive T cell therapy - tebentafusp

Tebentafusp is a bispecific agent built on the Immune-mobilizing Monoclonal T-cell receptor Against Cancer (ImmTAC) platform, combining a soluble T-cell receptor that recognizes a gp100-derived peptide presented by HLA-A02:01 with an anti-CD3 single-chain fragment. It has significantly extended overall survival in adults with previously untreated metastatic UM [37, 178–182]. The gp100 antigen (Pmel17 or ME20-M) is highly expressed in melanoma cells, minimally in normal melanocytes, and absent in non-melanocytic tissues [142]. *In vitro*, tebentafusp redirects CD8^+^ and CD4^+^ T cells to gp100^+^/HLA-A02:01^+^ melanoma lines, enhancing cytokine production including interleukin 2, interleukin 6, tumour necrosis factor-alpha (TNFα), and interferon-gamma (IFNγ) and cytolytic activity. TNFα and IFNγ promote tumour cell apoptosis, lymphocyte activation, and DC maturation [180, 181]. Its antitumor efficacy is restricted to gp100^+^/HLA-A*02:01^+^ tumours [180]. Although gp100 is expressed in CoM, tebentafusp has not yet been evaluated in this subtype [183]. Further studies should assess its clinical potential in CoM and strategies to overcome HLA restriction.

## Future perspectives and conclusion

Managing CoM presents a significant challenge due to its elevated recurrence and metastasis rates [2, 45]. However, recent advances in oncology have deepened our understanding of cancer biology, leading to the development of innovative therapies. Enhanced knowledge of the genetic, molecular, and immunological mechanisms underlying CoM pathogenesis has paved the way for novel treatment strategies, offering new hope for improved outcomes [2, 9, 45, 56].

Emerging therapeutic strategies for CoM include targeted molecular inhibitors, ICIs, and DC immunotherapy. Due to genetic similarities with cutaneous melanoma and other mucosal melanomas, treatments designed for these cancers are increasingly being applied to advanced or metastatic CoM, yielding promising results [26, 184–186]. Immunotherapy is being investigated for its potential benefits in cases with high TMB, either as a standalone treatment or combined with targeted therapies [9, 42, 43, 48, 49, 54, 155]. Furthermore, BRAF and MEK inhibitors, which target BRAF mutations and the activation of the MAPK pathway in CoM, have shown substantial benefits when combined [45, 187].

Preclinical research explores several novel therapeutic targets for CoM and CM, including c-KIT, ERK1/2, PI3K/AKT/mTOR, TERT, and EZH2 [32, 188–197]. While their effectiveness is still under evaluation and may not provide a universal solution, these targets could play a valuable role in personalised treatment strategies based on genetic screening, particularly for patients without BRAF or those with rare KIT mutations [1, 45].

ICIs can be used for all melanoma types, including cutaneous, mucosal, uveal, and conjunctival melanoma, though their efficacy varies based on genetic features. TMB is a key predictor of response, with higher TMB levels associated with better outcomes. Most clinical trials have focused on metastatic CM, often excluding patients with uveal and conjunctival melanoma, resulting in limited data for CoM, primarily from case reports and small series. Nevertheless, ICIs show promise for advanced CoM, with dosing regimens similar to those used for CM. Additionally, targeted molecular inhibitors targeting mutated intracellular mediators like BRAF and MEK have also demonstrated encouraging results [56].

Introducing new therapies has sparked renewed interest in SLNB and noninvasive testing for CoM. Research supports SLNB as a reliable staging tool for CoM, with sentinel node positivity strongly linked to lower overall survival rates. A positive SLNB signals a higher risk of systemic metastasis, highlighting the need for vigilant postoperative monitoring and potential adjuvant therapies. SLNB results can now lead to curative interventions, and early metastasis detection may improve the success of emerging treatments [104, 125, 198].

Managing CoM presents multiple challenges in prevention, diagnosis, treatment, and follow-up. Identifying patients who will benefit most from new therapies and optimising treatment choices are critical. Invasive tumour biopsies carry risks, highlighting the need for noninvasive diagnostic methods and real-time disease monitoring through biomarkers. In other cancers, noninvasive testing methods like circulating tumour cells (CTCs), circulating tumour DNA (ctDNA), cell-free DNA (cfDNA), tumour-derived exosomes, tumour-educated platelets, and micro-RNA are employed for diagnostics and patient follow-up. These liquid biopsy techniques can use samples from plasma, urine, and potentially tears in the case of CoM [96].

Despite promising outcomes from targeted therapies and ICIs, clinicians must also consider their potential specific adverse events, which can affect multiple organ systems. As in any clinical decision-making process, these factors should be thoroughly considered in the treatment decision-making process [1, 2, 45].

Addressing treatment resistance is crucial, especially since it frequently occurs in patients who initially have positive responses. Some researchers suggest exploring combinations of BRAF and MEK inhibitors, AKT pathway-targeting drugs, YAP1 inhibitors, PD-1/PD-L1, and CTLA-4 inhibitors [45]. Additionally, the combination of PD-1/PD-L1 and CTLA4 inhibition should also be investigated. Adding immune stimulatory agents like IFN-alpha, already used in ocular tumor treatments, shows promise. INF-alpha, available as eye drops or for intralesional application, is already employed in the localised therapy of malignant tumours on the ocular surface [199, 200]. Combining immunotherapy with radiotherapy or photodynamic therapy may enhance immune responses in patients with metastatic or advanced CoM [1, 2, 45].

Further research is essential to clarify the pathogenesis of CoM, particularly the distinctions between sun-exposed and non-sun-exposed lesions. It is crucial to explore the roles of underlying lesions, melanin pigments, and the immune system in the transformation of melanocytes. Investigating whether CoM behaves consistently across diverse populations is important, as most existing studies focus on North American and European cohorts. Additionally, examining variations in the genetic profiles of CoM among different populations is warranted. Given its rarity, international collaboration and including CoM patients in cutaneous and mucosal melanoma trials is crucial, along with maintaining proper registries for comprehensive data evaluation [1, 6, 45].

The predictive significance of genetic alterations in CoM is not yet fully understood, making prognostic genomic analysis uncommon in their management. As genomic analysis becomes more accessible, molecular profiling of these tumours, even in localised stages, will improve our understanding of their biological behaviour and progression. This will enable personalised treatment strategies and enhanced monitoring for patients with high-risk genetic features [45]. Future research should focus on uncovering the genetic background of CoM and evaluating the roles of genetics and epigenetics in tumour behaviour. Key areas of investigation include differentiating between benign and malignant lesions, identifying those at high risk of recurrence or metastasis, and selecting the most suitable therapies for patients. A major challenge lies in identifying the molecular drivers of these alterations to achieve clinically significant therapeutic outcomes in patients with CoM [22]. Rapid advancements in sequencing techniques will facilitate this process, and integrating tumour genomic analysis into the standard clinical management of CoM could enhance and personalise treatment for this aggressive cancer.
